# A comprehensive review of capsaicin: Biosynthesis, industrial productions, processing to applications, and clinical uses

**DOI:** 10.1016/j.heliyon.2024.e39721

**Published:** 2024-10-24

**Authors:** Anoth Maharjan, Bala Murali Krishna Vasamsetti, Jung-Ho Park

**Affiliations:** a Bio-Evaluation Center, Korea Research Institute of Bioscience and Biotechnology, Cheongju, 28116, Republic of Korea; b Toxicity and Risk Assessment Division, Department of Agro-Food Safety and Crop Protection, National Institute of Agricultural Sciences, Rural Development Administration, Wanju-gun, 55365, Republic of Korea; c Department of Applied Biological Engineering, KRIBB School of Biotechnology, Korea University of Science and Technology (UST), 217 Gajeong-ro, Yuseong-gu, Daejeon, Republic of Korea

**Keywords:** Capsaicin, Anti-oxidant, Pharmacological effects, Industrial production

## Abstract

Capsaicin, the main bioactive compound in chili peppers, is widely known for its diverse pharmacological effects, including antioxidant, anti-inflammatory, and anticancer effects. Despite its therapeutic potential, the low yield of natural capsaicin and the challenges in producing it on a large-scale limit broader industrial and clinical applications. This review provides a comprehensive analysis of capsaicin's biosynthesis in plants, chemical and enzymatic synthesis methods, and recent advancements in green production technologies. In addition, innovative applications such as drug delivery systems using nanoencapsulation and micelles are being developed to improve the bioavailability and therapeutic efficacy of capsaicin. Key findings highlight the use of capsaicin in food preservation, packaging, and pharmaceutical formulations. Future research should prioritize the refinement of synthetic routes, innovative delivery technologies, and the development of sustainable industrial processes to fully exploit the therapeutic and commercial potential of capsaicin.

## Highlights

• Capsaicin has antioxidant, anti-inflammatory, and anti-cancer properties.
• Industrial uses include traditional and novel technologies like nanotechnology for better drug delivery.
• Environmentally friendly synthesis methods reduce environmental impact while meeting rising demand
• Capsaicin’s interaction with TRPV1 receptors supports its potential in pain relief, obesity control, and cardiovascular health.

## Graphical abstract

**Figure:**
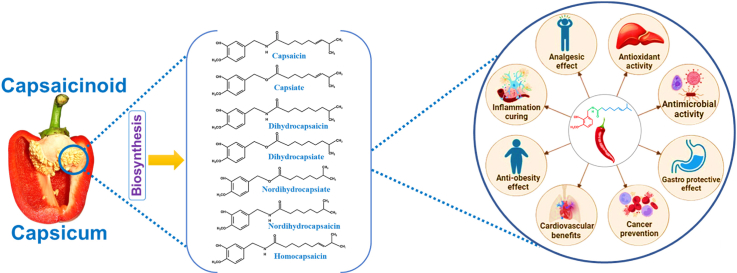

## Abbreviations

Pun1: Pungent gene 1
AT3: Antithrombin 3
Csy1: Citrate synthase 1
C/EBPα: CCAAT/enhancer binding protein
VR1: Vanilloid receptor 1
TRPV1: Transient receptor potential vanilloid 1
TPA: Tetradecanoylphorbol-13 acetate
HCT116: Human colorectal carcinoma cell 116
NADH: Nicotinamide adenine dinucleotide hydrogen
tNOX: Tumor-associated NADH oxidase
AMPK: AMP-activated protein kinase
NOTCH: Neurogenic locus notch homolog protein
UCP2/UCP3: Uncoupling protein 2/3
PPARγ: Peroxisome proliferator-activated receptor γ
GLP-1: Glucagon-like peptide-1
WAT: White adipose tissue
NQO1: (NAD(P)H Quinone Dehydrogenase 1)
C4H: Cinnamic acid-4-hydroxylase
PAL: Phenylalanine ammonia-lyase
CAOMT: Caffeic acid-o methytranferase
QTL: Quantitative Trait Locus
SLN: Solid lipid nanoparticles
THF: Tetrahydrofuran
AVAM: Acylvanillamines
BHT: Butylated hydroxytoluene
NLC: Nanostructured lipid carriers
SD: Sprague Dawley
DPD: Dissipative particle dynamics
BRL-3A: Buffalo rat liver

## 1 Introduction

Capsaicin, a widely recognized member of the vanilloid family, has drawn a significant attention in the scientific community because of its broad spectrum of pharmacological effects and diverse bioactive characteristics [1]. This potent compound, which originates from chili peppers, has applications in pain management, cardiovascular health, anti-inflammatory, and metabolic regulation. On top of that, *Capsicum frutescens* is an important source of novel antioxidants and antibacterial compounds, in addition to being used as a flavoring and coloring agent [2]. In addition to their macro- and micronutrient content, pepper varieties possess a range of bioactive compounds with unique functional and technical characteristics that are highly beneficial economically [2].

Capsaicin, a naturally occurring alkaloid extracted from the fruits of the Capsicum plant, is chemically known as trans-8-methyl-N-vanillyl-6-nonenamide. It belongs to the vanilloid family, which also includes zingerone (derived from ginger), eugenol (derived from bay leaves), and vanillin (derived from vanilla). The biological effects of vanilloids, including capsaicin, are primarily attributed to the presence of a vanillyl group (4-hydroxy-3-methoxybenzyl). Structurally, capsaicin features a benzene ring and a long hydrophobic carbon tail with a polar amide group, making it fat-soluble but water-insoluble. With a melting point of 62–65 °C and a molecular weight of 305.4 kDa, capsaicin is an off-white, fat-soluble, odorless, and spicy-smelling solid. Its solubility in alcohols and other organic solvents makes it suitable for use in topical treatments and sprays [[3], [4], [5]].

Plants are capable of synthesizing a diverse range of organic chemicals, including secondary metabolites that play ecological roles such as attracting pollinators, adapting to environmental stress, and defending against predators, insects, and pathogens [[6], [7], [8]]. One such secondary metabolite is capsaicin, produced by chili fruits as a defense mechanism against predators, with approximately 89 % of its concentration found in the placenta and only 5–6% in the pericarp [9]. The synthesis of capsaicin involves the combination of vanillylamine, derived from the phenylpropenoid pathway, with a short, branched fatty acyl chain from a branched amino acid [9]. Although multiple genes are involved in capsaicin biosynthesis, their precise roles remain largely unknown. For example, the *Pun1* gene codes for the antithrombin 3 (AT3) protein, which contributes to pungency, while *Csy1* encodes capsaicin synthase, an enzyme crucial for the final biosynthetic step [10].

Recent research has expanded our knowledge of capsaicin's diverse uses in food, medicine, and its evolutionary significance in Capsicum species [10]. In humans, capsaicin binds to transient receptor potential vanilloid subtype 1 (TRPV1) receptors in peripheral nerve fibers that detect pain. This binding increases calcium levels within cells, triggering the release of neuropeptides and transmitting neural signals that perceive excessive heat, which can induce an inflammatory response upon intense exposure [1,[11], [12], [13]].

The main pungent component of Capsicum spp. capsaicin is produced and accumulated in the placental tissues of the fruit, which not only enhances the flavor but also the pungency of food [14,15]. This dual role underscores capsaicin's potential benefits in both the food and pharmaceutical industries. Numerous studies have therefore focused on optimizing capsaicin production through chili plant cultivation, chemical synthesis, enzymatic synthesis, and cell or tissue culture techniques. To date, studies have demonstrated the diverse biological and physiological properties e.g., as an antioxidant, antimicrobial [16], anti-inflammatory [17], anti-cancer [18], anti-tumor [19], anti-obesity [20], cardio-protective [21], gastro-protective [22], and metabolic modulating [23]. However, despite its promising therapeutic properties, the industrial production and large-scale application of capsaicin remain limited due to challenges in synthesis, cost, and safety regulations.

Thus, the aim of this review is to provide a comprehensive examination of the biological synthesis of capsaicin in plants, explore current industrial production methods, and evaluate its potential applications and clinical uses. By synthesizing existing literature and highlighting recent research advances, we aim to elucidate mechanisms of action, address safety concerns, and highlight the value of capsaicin's as a versatile therapeutic agent in both the food and pharmaceutical industries.

## 2 Production and synthesis of capsaicin

Capsaicin is an alkaloid found specifically in the fruit of the Capsicum plant and is the main component responsible for the spicy taste. The placental tissues that store the seeds in the fruits contain a significant amount of capsaicin, which is likely why it serves as a deterrent to herbivores.

### 2.1 Biosynthesis of capsaicin in plants

There are two well-defined pathways for involved in capsaicin biosynthesis in plants. One The first pathway is the phenylpropanoid pathway which determines phenolic structure, and second is fatty acid metabolism, where fatty acid molecules are synthesized was determined [24]. Phenylalanine and either valine or leucine serve as primary precursors for the phenylpropanoid and fatty acid pathways, respectively, both of which contribute to capsaicin biosynthesis. Capsaicin is synthesized and accumulates in the vesicles of epidermal cells in the placental tissue, where it contributes to the pungent odor of the fruit. During fruit development, capsaicin concentration gradually increases and reaches its maximum after 40–50 days [25], leading to degradation into secondary compounds due to antioxidant activity [26].

**Fig. 1:**
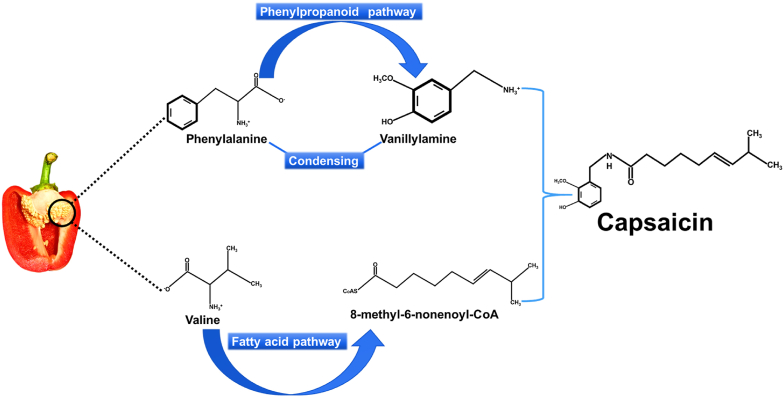
Biosynthesis of capsaicin (modified from Stewart et al.).

The biosynthesis of capsaicin involves numerous enzymes, many of which remain poorly understood, making the control of these pathways unclear. Four key enzymes are involved in this process: phenylalanine ammonia-lyase (PAL), which initiates the formation of cinnamic acid in the phenylpropanoid pathway [27] as well as, cinnamic acid-4-hydroxylase (C4H), ρ-coumaric acid-3-hydroxylase (C3H), and caffeic acid-o methytranferase (CAOMT), which together help to increase the capsaicin concentration [28]. Through a series of enzymatic steps, the fatty acid synthesis pathway produces 8-methyl-6-CoA, a precursor in branched fatty acid synthesis. After formation of two branched chains, vanillyamine and 8-methyl-6-nonenoyl-CoA are eventually transformed into capsaicin by acyl-transferase (AT) (Fig. 1) [10].

While the complete biosynthesis of capsaicin is still hypothetical, researchers remain keenly interested in understanding the process [29]. Several studies have aimed to increase or enhance the production of pungent compounds, as water stress has been found to affect the phenylpropanoid pathway [30]. Research shows also indicate that 8-methylnoneic acid produced in lower quantities than vanillylamine, making it the limiting substrate for capsaicin production when capsaicin precursors are administered [31].

Understanding capsaicin synthesis also involves measuring its pungency, historically gauged using the Pepper Scoville Heat Scale. Developed by Wilbur Scoville in 1912, this scale quantitatively measures the spiciness of chili peppers by assessing capsaicin content [32]. It ranges from 0 to 16 million Scoville heat units (SHU), with pure capsaicin at the highest rating. The original method required diluting a chili pepper extract with sugar water until the heat was imperceptible to a panel of tasters, with the Scoville rating determined by the necessary dilution factor. For example, a pepper rated at 100,000 SHU necessitates a dilution of 1 part chili extract to 100,000 parts water to neutralize the heat sensation [33]. The pungency of peppers is categorized into levels based on SHU: non-pungent (0–700 SHU), mildly pungent (700-3000 SHU), moderately pungent (3000–25,000 SHU), highly pungent (25,000–70,000 SHU), and very highly pungent (above 80,000 SHU) [34]. Although modern assessments typically employ more consistent and accurate chromatographic methods, understanding the traditional Scoville scale provides context for these more recent technologies [35].

### 2.2 Genetic control of capsaicin production

Despite capsaicin's significant commercial and agricultural importance, there is limited information about the genetics behind its production. Genetic and environmental factors cause considerable variation in capsaicin accumulation among chili genotypes [36]. The first genetic study of capsaicin accumulation was carried out using molecular mapping, and it found a Quantitative Trait Locus (QTL) named “cap” (capsaicin), which may help to increase the level of pungency [37]. Many genes associated with capsaicinoid production are linked to biosynthesis, though little is known about the specific location and function of the genes regulating capsaicin accumulation.

The genes *Pal*, *Ca4h* and *Comt* encodes phenylpropanoid pathway [38] and genes *Kas*, *Acl* and *Fat* encodes in fatty acid metabolism [39]. Spiciness is a dominant feature and is linked to the *Pun1* locus, which encodes acyltransferase AT3, expressed specifically in the placenta of pungent genotypes. The highest expression of *Pun1* gene correlates with the peak accumulation of capsaicinoids, determining the occurrence of the pungent phenotype [10].

Initially, AT3 was thought to be associated with capsaicin synthase (CS), but subsequent research identified the *Csy1* gene as encoding CS, and the sequence differences confirmed that *Pun1* does not code for CS [40]. Later studies revealed that the product of *Pun1* is involved in the formation of vesicles, where capsaicinoids accumulate, rather than in the direct synthesis of these compounds [41]. The development of these vesicles is critical for the expression of the pungent phenotype.

### 2.3 Chemically and enzymatically synthesis of capsaicin

Interest in capsaicin has surged due to its various properties and potential medical applications [42]. Numerous research projects have been conducted to study the synthesis of natural capsaicin and synthetic variants with properties similar to natural capsaicin [43]. Compared to chemical synthesis, enzyme-catalyzed synthesis offers advantages such as the use of non-toxic reagents and greater substrate specificity [44].

**Fig. 2:**
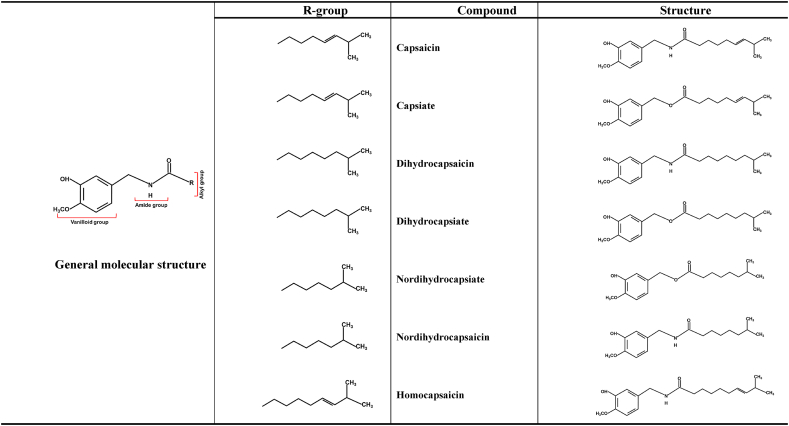
Various amines and donors as substrates for the synthesis of analogs of capsaicin.

Kobata and colleagues conducted several studies aimed at producing capsaicin analogs through amidation processes using various lipases as catalysts [45]. By using vanillylamine in combination with fatty acid derivatives as substrates in the oleose phase, they were able to achieve a capsaicin yield of about 40–59 % [46]. Various non-spicy analogs were synthesized by altering the acyl chain lengths and aromatic ring substitutions. These efforts produced two pungent analogs and several weakly pungent ones, all of which have potential applications (Fig. 2).

### 2.4 In-vitro synthesis

The *in-vitro* synthesis of capsaicin and its analogs represents a promising alternative for increasing capsaicin production. The incorporation of precursors and intermediates from the biosynthetic pathway, such as phenylalanine, ferulic acid and vanillylamine, has shown promising results when applied to cell or tissue cultures [47]. Here are a few of the steps encompassed in the *in-vitro* synthesis of capsaicin.

#### 2.4.1 Creating cell suspension cultures and inducing calluses from chili fruits

The first step in the *in-vitro* synthesis of capsaicin involves the induction of callus formation. Chili fruit explants are placed in a plant tissue culture medium containing a high concentration of auxin and a low concentration of cytokinin, which promotes callus development [48]. The placental tissue of chili fruits is typically used as an explant because it contains about 90 % of the capsaicin content of the chili.

#### 2.4.2 Selection of cell lines with high yields

The crucial step in capsaicin production is selecting cell lines that produce a high amount of capsaicin to maximize overall production. It is important to examine callus from different parent plants and examine differences in biochemical activity between clonal cells to identify those with the highest capsaicin production. Techniques like mutation and selective agents can be employed to identify and isolate cell lines that yield the maximum amount of capsaicin. In the presence of cytotoxic inhibitors or under environmental stress, only resistant cells will proliferate [49].

#### 2.4.3 Immobilization of plant cells for the synthesis of capsaicin

A modern approach to enhancing the production of secondary metabolites involves the use of Surface Immobilized Plant Cells (SPIC). Immobilization of plant cells, in this case, leads to significantly higher yields of capsaicin compared to freely suspended cells [50]. Immobilized Capsicum cell cultures have been found to produce many times more capsaicin than non-immobilized cultures [51]. The immobilization technique offers advantages such as increased yields of stable products, longer cell viability, and reduced production costs in plant cell culture [52].

#### 2.4.4 Elicitation of culture to enhance overall capsaicin output

Elicitation is a technique used to increase the production of specific compounds in cultured cells. Elicitors can be biotic (biological in origin) or abiotic (non-biological) and can come from either endogenous or exogenous sources. Abiotic elicitors include metal ions such as Cu^2^⁺ and Cd^2^⁺, and factors such as pH, while biotic elicitors include molecules like pectin or cellulose from plant cell walls and chitin or glucans from microorganisms (Table 1) [53]. Additionally, exogenous triggers can include polysaccharides, polyamines, and fatty acids synthesized in the extracellular environment, whereas endogenous triggers are derived from the cells themselves (Table 2) [53].

**Table 1:** Biotic and abiotic elicitors.

Biotic elicitors	Abiotic elicitors
1. Produced by microorganisms and recognized by plant cells (enzymes)	1. Physical or chemical in nature.
2. Produced on the plant cell wall by the action of microbes (fragments of pectin)	2. Uv light
3. Formed as a result of plant enzyme action on microbial cell walls (chitosans, glucans)	3. Denatured proteins (RNase)
4. Substance produced by plants in response to an outer agent	4. Frequent freezing-thawing cycles.
	5. Unnecessary media components.
	6. Chemicals that has high DNA affinity.
	7. Detergents
	8. Fungicides
	9. Herbicides.

**Table 2:** Elicitors on the depending upon origin.

Exogenous elicitors	Endogenous elicitors
1. Formed around outer membrane of the cell	1. Induced by biotic or abiotic signals that are formed within the cell via secondary reactions
2. Chitosan, Glucans, glucomannose as polysaccharides	2. Hepta-beta-glucosides
3. Polyamines, Glycoproteins as polycations	3. Oligomers such as Alginate.
4. Cellulase, polygalacturonase, etc. as cellular enzymes:	
5. Arachidonic acid, Eicosapentnoic acid as fatty acids:	

This comprehensive approach to *in-vitro* capsaicin synthesis holds significant potential for improving capsaicin yields, offering a controlled environment for studying and manipulating the biosynthesis process.

## 3 Industrial production of capsaicin

The industrial production of capsaicin has been explored through several synthetic pathways, each highlighting the key reactions involved in introducing the double bond at the C6 position of capsaicin's side chain. Industrially, capsaicin and its analogs are synthesized using amines and chlorinated fatty acids at temperatures ranging from 140 °C to 170 °C and under moderate pressure [54]. The Choi and Yoon group used bio-isosterism to generate a 1-hydroxy-2-pyridone analogue of capsaicin with similar biological effects [55]. Another study investigated reactions between vanillyl alcohol and nonanoic acid in tetrahydrofuran as the reaction medium, using equimolar amounts of di-isopropyl azodicarboxylate and triphenylphosphine, yielding 67 % vanillyl nonanoate at room temperature after 24 h [56]. Similarly, another process utilized cerium (III) chlorate as a catalyst for the selective esterification of phenolic alcohols, resulting in a 70 % yield of vanillyl nonanoate [57].

The non-pungent analogue dihydronorcapsaicin β-D-glucopyranoside has also been synthesized through combined chemical and enzymatic methods. Enzymatic synthesis presents an attractive substitute for conventional chemical synthesis, as the toxicity of the necessary chemicals limits the success of chemical synthesis of capsaicin. capsiate, dihydrocapsiate, and nordihydrocapsiate, were identified from the non-pungent red pepper variety CH-19. Capsinoids differ from capsaicinoids mainly by their ester moiety, whereas capsaicinoids possess an amide moiety. Despite this difference in linkage, capsinoids and capsaicinoids exhibit notable structural similarities [25].

### 3.1 Mechanism of action of capsicum component on industrial scale

Industrial production of capsaicin holds great potential, but further improvements could increase production efficiency [58]. For example, modifications can be made to the amide region and the vanillyl moiety of capsaicin through methodological approaches [59]. Capsaicin offers significant conformational flexibility, with six torsion angles that can be adjusted across a 0–360° range, resulting in a large variety of geometries [60]. Some studies suggest that the lipophilic domain of the capsaicin receptor may bind polar groups, while other structural components may help preorganize the lipophilic acyl moiety of N-acylvanillamines (AVAM) for binding [61]. For example, long-chain capsaicin analogs such as N-stearoylvanillamine are inactive, but the introduction of unsaturations such as N-oleylvanillamine (Olvanil) restores activity, highlighting the importance of molecular modifications [56].

Capsiate, a non-pungent component of bell peppers known for its NF-κB inhibiting and fat-burning properties, has a simpler equivalent in vanillyl ester [62,63]. Its synthesis from vanillol has been used to integrate prove its relevance by benchmarking the impact of several Lewis acids and acylating reagents on the esterification reactions. Using tetrahydrofuran (THF) as a solvent and an equimolecular ratio of alcohol and acylating agent (chloride, anhydride), cerium (III) chloride, indium (III) chloride, and ytterbium (III) triflate were examined (at 20 % loading) as promoters in a first set of experiment. Vanillol's acylation with nonanoyl chloride was ineffective with indium (III) chloride, but ytterbium (III) triflate successfully produced a mixture of phenyl and alkyl esters [57].

Nordihydrocapsiate was the only reaction product that cerium (III) chloride, yielding 53 %. In comparison, nonanoic anhydride produced only 15 % conversion. However, reducing the lanthanide salt load to 0.5 % significantly improved the yield, increasing it to 70 %. This improvement was likely due to reduced degradation of an unstable pro-quinoid compound in the reaction mixture [64]. This optimized procedure was used later applied to the synthesize other fatty esters of vanillol compounds, with yields comparable to or even surpassing those obtained from Mitsunobu esterification [57].

## 4 Benefits and clinical uses

Capsaicin possesses potential significance in the food and pharmaceutical sectors. As a result, numerous researches are being conducted to increase its production by adapting to the circumstances [65]. Its pungent taste, which is a natural defense mechanism against fungi and herbivores, has led to extensive research into its effects on the human body for over a century [66].

**Fig. 3:**
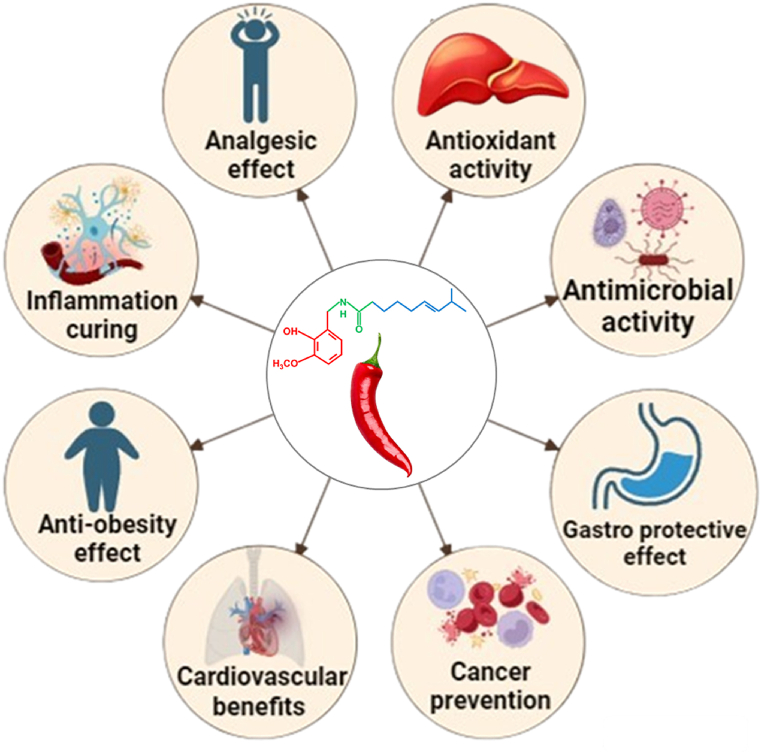
Health benefits of capsaicin.

One of the earliest studies, conducted by Hogyes in 1878, demonstrated that the topical application of pepper extract on human skin caused a burning sensation and hyperemia [67]. Later studies using animal models showed that intravenous injection of capsaicin extract led to a decrease in blood pressure, an increase in saliva and stomach production, and an increase in intestinal activity [68]. Capsaicin has thus proven to be an interesting pharmacological agent and research is underway to determine its applicability in various clinical conditions (Fig. 3 and Table 3) [65].

**Table 3:** Summary of the pharmacological benefits and its mechanism of action.

Health beneficial	Mechanism of action	Reference
Analgesic effect	vlPAG leads to TRPV1 activation which helps to release glutamate into RVM and induce the activation of OFF-cells and reduced the pain pathway	[213]
Antioxidant activity	Phytochemical components - phenolics and flavonoids inhibits radiation-induced biochemical alterations including protein oxidation and lipid peroxidation	[214]
Antimicrobial activity	Disrupt the peptidoglycan structure due to lipid-lipid interactions providing liquidity in the cell wall and also induced the osmotic stress, responsible for gene expression inhibition	[215]
Gastro protective effects	Due to activation of TRPV1 at gastric sensory neurons – stimulates the release of CGRP and NO	[216]
Cancer prevention	ROS generation, increased in intracellular Ca^2+^, activation of transcription factors (NF-κB and STATs), disruption of mitochondrial membrane transition potential, and AMP-dependent kinase pathway	[217]
Cardiovascular benefits	Due to presence of TRPV1 in platelets provoked Ca^2+^ release from intracellular platelet contributing to ADP and thrombin induced platelet activation	[218]
Anti-obesity effect	Lowered fasting glucose, insulin, leptin level and also lower the tumor necrosis factor-alpha (TNFα), monocyte chemo-attractant protein-1 MCP-1), interleukin (IL)-6 mRNAs in adipose tissue and liver.	[219]
Inflammation curing	Inhibits the NF-κB phosphorylation which then reduces downstream IL-6 and TNFα target.	[220]

vlPAG: ventrolateral periaqueductal gray; TRPV1: transient receptor potential vanilloid 1; RVM: rostroventral medulla; CGRP: calcitonin gene-related peptides; NO: nitric oxide; ROS: reactive oxygen species; NF-κB: nuclear factor κ; STATs: signal transducers and activators of transcription; AMP: Adenosine monophosphate; TNFα: tumor necrosis factor-alpha; MCP-1: monocyte chemo-attractant protein-1; IL-6: interleukin-6.

## 5 Industrial benefits

### 5.1 Application in food industry

Capsaicin serves as a key component in multiple sectors, including self-defense, pharmaceuticals, cosmetics, insect repellents, and oral herbal supplements. Due to its numerous benefits, capsaicin is widely consumed in several countries. Chili peppers not only serve as a popular spice, but also add spice and flavor to dishes. For example, daily chili consumption in countries such as India, Thailand, Saudi Arabia and Mexico has been estimated to be around 2.5, 5.0, 15.5 and 20.0 g per person, respectively [69]. Some people are unable to consume spicy foods due to the burning sensation and irritability of capsaicin [70]. However, the technological and practical advantages of capsaicin helped expand its range of applications (Table 4).

**Table 4:**
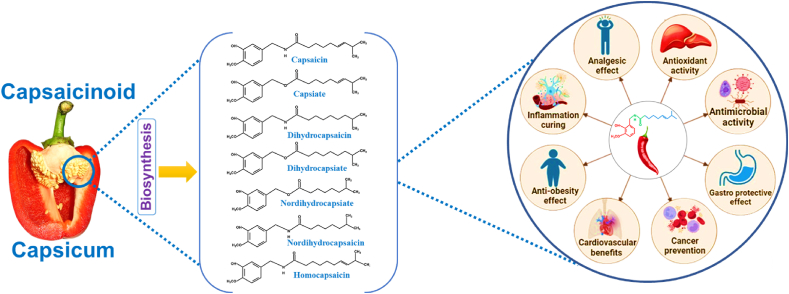
Summary of the uses of Capsaicin on food application.

Category	Application type	Functions	References
Flavor	Gochujang (chili pepper)	Hot-flavored instant noodles	[221]
	White pepper	Vegetable soup	[222]
	Pepper paste	Chocolate-flavored yogurt with menthol	[75,222]
	Solid pepper	Cheese-flavored waffle	[222] [75,76]
	Hot pepper sauce and hot ketchup	kimchi	
	Hot pepper sauce and hot ketchup Minced chili powder	Quick boodles	
		Spicy paneer cheese	[74]
		Pork patties	[77]
	Pepper oleoresin	Boosts saltiness	[78]
	Chili	Strawberry flavor	[80]
	Chili pepper powder	Sweetness of tomato soup	[83]
		Starch gruels	[83] [84]
		Sauces	[83,84,91]
	Hot chili sauces	Grilled pork meat	
	Red hot pepper extracts	Flavor and coloring agent	
Preservation	Bell pepper (*Capsicum annuum* L)	Managing foodborne pathogens and mitigating product spoilage	[58]
	Microcapsule	Antioxidant and antimicrobial	[85]
	Fabricated capsaicin with chitosan	Quorum sensing inhibitory effect	[93]
	Piperine	Antifungal	[86]
Packaging film	Ethyl cellulose composite film incorporated with capsaicin	Softness, waterproof, and eco-friendly	[94]

#### 5.1.1 Flavoring of food

Due to its pungency, capsaicin is a common spice, culinary flavoring, and seasoning used throughout the world. Capsaicin is found in various forms, such as paprika, chili powder [71], and red pepper paste sauces [72], including hot ketchup and hot pepper sauces [71,73]. Along with these dishes, spicy paneer cheese [74], many varieties of kimchi (such as radish and cabbage kimchi), and quick noodles [75,76] all include capsaicin. For instance, Reinbach et al. (2007) examined the correlation among oral burn, meat flavor, and instrumental texture in pork patties that had additional trigeminal stimuli, such as minced chili and chili powder. They figured out that while changes to the pork patties' texture had no effect on either burn or meat flavor intensity, the intensity of meat flavor decreased as burn intensity increased [77].

A study by Narukawa et al. (2011) showed that adding capsaicin to NaCl solutions significantly boosts the perceived saltiness [78]. Similarly, Wang et al. (2022) found that adding capsaicin either by alone or in conjunction with pepper oleoresin similarly enhances the saltiness of NaCl [79]. In a different investigation, Prescott and Stevenson et al. (1995) looked at how capsaicin affected retronasal olfaction in strawberry flavor, but they didn't find any proof that capsaicin lessens the strength of the flavor [80]. Despite the wealth of research on cross modal interactions between trigeminal and taste, smell, flavor, and texture perception using simplified model stimuli, there is a surprising lack of studies investigating these interactions in complex food systems or commercially available products [81].

Prescott et al. (1993) found that capsaicin decreased the sweetness of tomato soups, while also measuring their saltiness, oral burn, and overall intensity, although they did not evaluate flavor and mouth-feel perception [82]. Furthermore, Kostyra et al. (2010) studied the effects of incorporating chili pepper powder and capsaicin into a variety of liquid foods, including starch gruels, soups, sauces, and water solutions. They discovered that the type of carrier and its complexity had a significant impact on the severity of the burn caused the capsaicin caused in the mouth. However, the effect of oral burn on flavor and texture perception was not determined [83]. Similarly, Djekic et al. (2021)examined the dynamic burn intensities of grilled pork meats coated with three types of hot sauces, suggesting that the intensity and duration of pungency sensations may be related to the type of sauce used [84].

#### 5.1.2 Preservation of food

Encapsulated capsaicin is used in long-term food preservation due to its release properties as an antioxidant and antibacterial agent, especially in the form of capsaicin microcapsules [85]. In addition, capsaicin can lower the amount of aflatoxin produced by *Aspergillus parasiticus* and is utilized as a food preservative when subjected to mycotoxin contamination [86]. Capsaicin contributes to the suppression of the ochratoxin A production nearly by 29–78 % in four *Aspergillus* group Nigri strains and about 61.5 % in *Aspergillus carbonarius* [87]. The study of antioxidant activity of capsaicinoids in canola oil at the temperatures of 60, 90, 12, and 180 °C was observed with the reduction of α-linolenic acid and linoleic acid and the oxygen consumption [88]. Antioxidant activity of capsaicin is close to or to some extent weaker than butylated hydroxytoluene (BHT) at 90, 120, and 180 °C [89].

#### 5.1.3 Functional foods

Capsaicin-infused noodles can retain the spice for a longer period and release it into the gastrointestinal system in a controlled manner [90]. Since most Asians like to slurp their noodles rather than chew them, layering the noodles will prevent the consumers from experiencing undue discomfort when eating. A variety of goods are made with capsaicin additives, such as yogurt that has been enhanced with the spice and fortified with oleic sunflower oil to give it a fresh flavor and a higher nutritional content [91]. The addition of capsaicin to the yogurt makes the taste spicier with better quality and a finer texture compared to traditional yogurt.

#### 5.1.4 Packaging film manufacture

Capsaicin also used to manufacture the transparent edible films made from sodium alginate. For example, composite films coated with fresh apple cubes blended with capsaicin keep the fruit fresh for a longer period [92]. Similarly, the chitosan films incorporated with different amount of capsaicin (0.3, 0.6, and 1.2 mg) results in the improvement of the biological, physicochemical, and mechanical properties. Further, ascapsaicin concentration increase, the anti-quorum sensing, antimicrobial, transparency, elasticity, hydrophobicity, and antioxidant properties of films can also be improve [93].

**Fig. 4:**
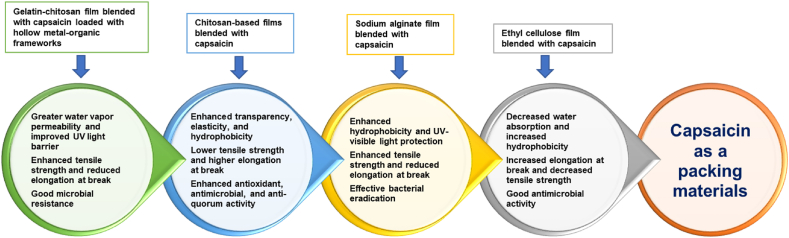
The effect of packaging film incorporated with capsaicin.

Additionally, gelatin/chitosan composite film blended with capsaicin hollow metal-organic frameworks are used as antibacterial biomaterials for food packaging. Moreover, Fe (III)-doped hollow framework (Cap-Fe (III) HMOF-5) used as capsaicin nanocarriers, regulating its hydrophobicity solving the phase separation problem. Continuing, the ethyl cellulose composite film incorporated with capsaicin exhibiting the transparency, softness, waterproof, and eco-friendly nature which serve as good antimicrobial activity against *Escherichia coli* and *Staphylococcus aureus*. Also, ethyl cellulose-capsaicin film effectively slows the fruit ripening process of bell peppers, keeping the fruits relatively fresh [94]. Thus, capsaicin incorporated film can be used for the food packaging materials prolonged their shelf life, as summarized in Fig. 4 [89].

### 5.2 Novel technologies involved capsaicin such as drug delivery or nanosupun based delivery in food usages

Capsaicin, known for its bioactivity, influences multiple signaling pathways and gene regulation through TRPV1-dependent and independent mechanisms. It has been shown to provide a variety of health benefits, including reducing blood sugar, promoting lipid metabolism, cancer inhibition, pain relief, gut health improvement, and anti-inflammatory effects [95]. Despite these promising properties, capsaicin's low solubility in water and its tendency to cause oral discomfort, such as burning sensations and stomach irritation, limit its effectiveness when consumed orally. To overcome these challenges, researchers have developed advanced delivery systems to improve capsaicin's bioavailability and reduce side effects [96].

#### 5.2.1 Liposomes, polymeric micelle, and micro/nano emulsions

Liposomal delivery systems are particularly effective at enhancing the bioavailability of capsaicin by improving its solubility and protecting it from degradation in the digestive tract. In a pharmacokinetic study involving male Sprague Dawley (SD) rats, the liposomal formulation showed improved solubility, reduced oral irritation, and enhanced bioavailability. Terrón-Mejía et al. (2018) developed a nanoliposome loaded with capsaicin, consisting of lecithin and chitosan, with a diameter of 18 nm [97]. To examine the chitosan coating of these nanoliposomes, a dissipative particle dynamics (DPD) approach was employed, revealing enhanced stability of the formulation. In a separate study, Giri et al. (2016) prepared capsaicin-loaded nanoliposomes with a mean particle size of 277.7 nm [98]. In animal experiments using male Swiss-albino rats, these capsaicin nanoliposomes demonstrated a protective effect against oxidative stress and tissue injury induced by sodium fluoride, suggesting that liposomes could be a promising delivery system for capsaicin [96].

Potential delivery systems for capsaicin intake have also been mentioned, including oil-water emulsions and liposomes laden with capsaicin. It was discovered that both formulations increased the body's absorption rate, membrane permeability, and bioavailability of capsaicin while also extending its dispersion time. The formulation is designed to target the maximum release of capsaicin in the colon, rather than the stomach, which is thought to be the optimal site for capsaicin absorption [99]. This approach reduces the risk of gastric irritation. In support of this, an in-vivo study conducted by Zhu et al. (2015) found no significant signs of gastric irritation in mice after consuming capsaicin in an oil-water emulsion [100].

Thin-film hydration-prepared polymeric micelles are another possible oral delivery method for capsaicin [101]. During encapsulation, capsaicin is solubilized within the micelle, which enhances its oral bioavailability and absorption rate due to its uniform and nano-sized particles. Although the maximum concentration of capsaicin-loaded micelles in rats is lower than that of free capsaicin, the micelle's longer circulation time translates to a longer reaction time and higher therapeutic efficacy. Zhu et al. (2014) implemented a thin-film dispersion technique to create a mixed polymeric micelle using phospholipids, sodium cholate, and polyvinylpyrrolidone that produced particles smaller than 50 nm [101]. The micellar system indicated to have better storage stability, improved in vitro release, and greater in vivo oral bioavailability of capsaicin after undergoing physiological and biological tests. Significantly, histological analysis of the stomach tissues in Sprague Dawley (SD) rats showed less gastrointestinal irritation, which can be related to the micellar system's extended circulation duration made possible by polyvinylpyrrolidone [96].

To improve the oral bioavailability of hydrophobic ingredients, microemulsion/nanoemulsion systems are being commonly utilized. These systems are generally created using high-pressure homogenizers, high-speed homogenizers, ultrasonicators, or microfluidizers in combination with emulsifiers and modified phase or solution conditions. For eg, Huang et al. (2008) explored the application of microemulsion methods to improve the transdermal distribution of the capsaicinoid nonivamide. They designed water as the exterior phase, isopropyl myristate as the oil phase, ethanol as a cosurfactant, and blended surfactant-based microemulsions made of Tween-80 and Span 20. According to the trials, these microemulsions improved nonivamide transdermal delivery by 3.7–7.1 times when compared to controls [102]. A nanoemulsion system was designed using the ultrasonication technique, wherein a capsaicin-loaded organogel served as the oil phase and Tween 80 as the emulsifier, resulting in a mean droplet size of 168 nm and a capsaicin loading capacity of 80.4 mg/mL [103]. Another stable nanoemulsion formulation was developed using oleic acid, labrasol, Tween 20, glycerol, and water through high-speed homogenization and ultrasonication [104]. This formulation demonstrated enhanced dissolution and low toxicity in studies. Furthermore, Han et al. (2020) successfully fabricated a capsaicin-loaded nanoemulsion by employing tocopheryl polyethylene glycol 1000 succinate as an emulsifier under high-pressure homogenization [105]. With a particle size of about 100 nm, a zeta potential of −36.4 mV, and an encapsulation effectiveness of 91.9 %, the assembled nanoemulsion illustrated improved stability and antioxidant qualities. Biological investigations confirmed that the emulsion was well absorbed by buffalo rat liver (BRL-3A) cells and caused less irritation to the gastrointestinal mucosa when compared to free capsaicin. This suggests that the emulsion has the potential to encapsulate and administer irritating nutraceuticals [96].

#### 5.2.2 Nanotechnology tools for delivery system

A number of benefits come with using delivery methods based on nanoparticles, such as their high loading capacity, stability, prolonged drug release, and potential to transfer hydrophilic and lipophilic nutraceuticals across biological barriers and cell membranes [[106], [107], [108]]. Many nanotechnologies have been used to increase the bioavailability of capsaicin. For instance, Contri et al. (2014) achieved an encapsulation efficiency of over 100 % by effectively encapsulating capsaicin and dihydrocapsaicin in polymeric nanocapsules [109]. Capsaicin was effectively encapsulated by Lv et al. (2017) utilizing folic acid-conjugated lipid nanoparticles that were prepared by a thin-film hydration method [110]. Remarkably, these nanoparticles extended the retention of capsaicin in the blood circulation of male SD rats and demonstrated an improved anti-cancer action in SKOV-3 ovarian cancer cells [111]. Further, Feng et al. (2018) added water to ethanol-dissolved capsaicin while swirling the mixture mechanically to create self-assembled capsaicin prodrug nanoparticles [112]. The capsaicin nanoparticles showed better bioavailability, higher gastrointestinal acid stability, increased hypolipidemic effect and lower mucosal irritation in physiochemical and biological studies. The oral administration of capsaicin by the nanoparticle systems resulted in notable improvements in stability and bioavailability [96].

Another method involved adding chitosan hydrogel to capsaicin nanoparticles to increase the drug permeability through skin [113]. Interestingly, when compared to free capsaicin combined with chitosan hydrogel, the capsaicin-nanoparticles showed greater rates of adhesion and retention in human skin. The deeper layers of skin benefited from a more effective diffusion and delivery of capsaicin due to the enhanced retention in the epidermis and dermis [114]. Furthermore, Amruthraj et al. (2015) developed silver nanoparticles capped with capsaicin, which showed to be compatible with blood cells through hemagglutination tests [115]. In a separate study, Bejrapha et al. (2011) designed nanocapsules loaded with capsicum oleoresin in a gelatin matrix to study how freezing, with or without excipients, influences the durability of these nanocapsules during freeze-thawing and freeze-drying [116]. In contrast to capsaicin-containing solid lipid nanoparticles (SLNs), capsaicin-loaded nanostructured lipid carriers (NLCs) showed improved retention in the stratum corneum and greater penetration into the skin. Using a model of LACA mice, the pharmacological effectiveness of capsaicin-loaded NLCs was evaluated in vivo. Stearic acid was used to integrate the capsaicin-NLC into a gel formulation. The capsaicin-NLC gel was shown to have higher permeability and retention through mouse skin compared to traditional creams containing capsaicin. In addition, the analgesic efficacy of capsaicin-NLC gel was significantly greater compared to capsaicin-containing cream as determined by the Radiant Mouse Tail Flick test [117]. To achieve optimal bioavailability and pharmacological effects of capsaicin, further research is required to identify more compatible encapsulation materials. Furthermore, the use of novel encapsulated capsaicin formulations in combination with other drugs or dietary supplements requires further investigation to elucidate the underlying chemical interactions and mechanisms.

### 5.3 Utilization of pepper byproducts as sources of bioactive compounds

Pepper byproducts, including seeds, skins, and stems, are rich in a variety of bioactive compounds that hold significant industrial value. These by-products, which are often discarded in conventional processes, can be further used to obtain valuable compounds such as polyphenols, flavonoids, carotenoids and capsaicinoids [118,119].

Polyphenols, flavonoids, and carotenoids, found predominantly in pepper skins, seeds, and stems, are key bioactive compounds known for their antioxidant and antimicrobial properties [118]. In the food industry, polyphenols act as natural preservatives by preventing spoilage, while flavonoids serve as natural colorants in both food and cosmetics, offering an alternative to synthetic dyes [[120], [121], [122]]. Carotenoids, widely used in food additives and nutraceuticals, are potent antioxidants with health benefits and stability [123]. Capsaicinoids, primarily concentrated in seeds and placental tissues, play functional roles in boosting metabolism in functional foods, and are incorporated into pharmaceuticals as analgesics and anti-inflammatory agents, enhancing their therapeutic potential [124,125].

On an industrial scale, the mechanisms of action of these bioactive compounds involve advanced extraction techniques, such as solvent extraction, enzymatic processes, and green technologies like supercritical CO₂ extraction [58,126,127]. These methods optimize the recovery of active compounds from pepper byproducts, ensuring their stability and potency in end products. For instance, the antioxidant activity of polyphenols and carotenoids is retained through encapsulation technologies, which allow for controlled release in food packaging and functional food applications [128]. In cosmetics, flavonoids are incorporated into formulations using emulsification or nanotechnology, which ensures their bioavailability and enhances their colorant properties over time [129].

In food applications, capsaicinoids are encapsulated to control their release and maintain stability, particularly in functional foods where gradual delivery enhances metabolic benefits [89]. Similarly, in pharmaceuticals, microencapsulation techniques help ensure sustained release in topical treatments, maximizing the therapeutic effect while minimizing side effects [130]. These advanced mechanisms of action enable industries to effectively harness the full potential of Capsicum components, transforming byproducts into high-value materials that meet both functional and consumer demands [131].

By extracting these valuable compounds, industries can significantly reduce waste and contribute to a circular and sustainable production model. Reusing by-products not only reduces raw material costs and increases production efficiency, but also minimizes environmental impact by reducing landfill use and reducing the carbon footprint. The valorization of by-products offers new opportunities for product innovation in sectors such as food preservation, cosmetics and pharmaceuticals, and is in line with the growing demand for environmentally friendly, sustainable solutions.

### 5.4 Cosmetic benefits

Due to the current environmentally friendly behavior of consumers and industry, there is great interest in finding bioactive substances, plant raw materials or plant extracts as natural components (or excipients) for cosmetic and pharmaceutical purposes. Therefore, taking this into account, certain characteristics, such as physical ones (like color, flavor, texture, or permeation) and bioactivities (like antimicrobial and antioxidant), that can affect how well a product performs should be investigated and approved by the relevant competent authority [132]. Natural ingredients are more beneficial than synthetic preservatives, which could enhance the health properties of cosmetic and pharmaceutical products while minimizing the negative effects of contact allergies. The primary cosmetic industry product claims, such as the antiaging effect and wrinkle reduction by fending off free radicals and sun radiation, may also be supported by these plant chemicals. All of these applications of bioactive substances as nutraceuticals or cosmeceuticals must be comply with international standards for the quality of medicines, with the most pertinent expert groups being those associated with the European Pharmacopoeia (Ph. Eur.), the Japanese Pharmacopoeia (JP), and the United States Pharmacopoeia (USP) [133].

#### 5.4.1 Enhancement of physical properties

Paprika powder is rich in capsaicinoids; therefore, these bioactive substances are a great source of hues for the cosmetic and pharmaceutical industries. According to the European Food Safety Authority (EFSA) and Food and Drug Administration (FDA), these natural ingredients do not need their approval; however, they must be safe for the user when used following labeled or customary conditions, and manufacturers are legally responsible for the safety of their goods and ingredients. In this regard, capsaicin used as spice can induce the undesirable side effects when use as a cosmetic color, however, it is acceptable as food additive [134,135]. For example, paprika oleoresin is used as cosmetic colorants in the bath oils (Lusch Handmade Cosmetics, S.L. URL: https://de.lush.com/search/site/paprika), shampoo, soaps, shower gels, and many beauty products including eye make-up and lipsticks (e.g., Color Marker Inc.; URL: http://www.colormaker.com/natural-ingredients_paprika).

The bioactive chemicals from pepper by-products can be included in high concentrations up to 5 % (w/w) in cosmetic goods without having any hazardous effects by using hexane, ethanol, or vegetable oil extraction [136]. The capacity of pepper oleoresin to be dissolved in fats, oils, and lipids-a feature known as lipophilicity-gives producers a significant advantage over the usage of essential oils because essential oils lack this ability. Some other pigments like chlorophylls and polyphenols, mainly anthocyanins, which are capsaicin varieties could be used as colorants in therapeutic compounds (UV protectors and antioxidants) in pharmacy and cosmetic products [137]. In line with this, red pepper by-products were extracted with acetone and water to create a natural dye that could be used on woolen fibers to create colorful clothes and textiles with respectable antibacterial characteristics [138].

#### 5.4.2 Preservation and stability of product

Because of antioxidant properties of capsaicin, it is used in medical products to reduce the oxidation of active substances and excipients. This bioactive antioxidant potential is influenced by their composition, how the pharmaceutical product is processed, how it is packaged, and how it is formulated [58]. Dehydrated green pepper with high levels of vitamin C (135–240 mg/100 gDW) could be utilized as a preservative and antioxidant in the cosmetics business [139]. Additionally, carotenoids found in capsicum extracts including capsanthin, lutein, and zeaxanthin that were extracted using oil-soluble solvents could be employed as an active component in cosmetics for skin care. Incorporating them into lipoproteins or membranes could increase their bioactivity and stability [140].

Antimicrobial preservatives are used to stop or slow the growth of bacteria, fungi, and molds that pose a risk of disease or deterioration in pharmaceuticals and personal care items, which often have a shelf life of more than three years [141]. Together with other bioactive substances present in Capsicum extracts, cinnamic acid and p-coumaric acid have demonstrated robust antibacterial properties against *Listeria monocytogenes*, *Staphylococcus aureus*, *Salmonella typhimurium*, and *Bacillus cereus*. They have also exhibited the ability to inactivate or inhibit the growth of spoilage and pathogenic microorganisms in industrial products. While there is considerable potential in utilizing Capsicum derivatives as preservatives, their commercial adoption remains limited. This is primarily because synthetic preservatives are not only more cost-effective but also offer a broader spectrum of antibacterial and antifungal activity. Additionally, synthetic preservatives can be easily integrated into formulations alongside other ingredients, enhancing their appeal for commercial applications. Nonetheless, a growing consumer demand for products derived from natural plant materials may lead to an upsurge in the utilization of natural preservatives in the cosmetics industry in the coming years [142].

#### 5.4.3 Beauty and health products applications

Pepper-derived compounds are utilized in cosmeceuticals and nutricosmetics, primarily for their antioxidant and analgesic properties, both in dietary supplements and topically applied products [143,144]. The increasing demand for new therapeutic solutions to treat skin diseases highlights the importance of considering various factors that may influence the effectiveness of topical medicinal agents, such as their active ingredients, excipients, potential interactions between ingredients, galenic properties, formulation, site of application, and the condition of the skin or mucosal surfaces [145].

The European Union (EU) and FDA have approved capsaicin as a medication for the topical treatment of neuropathic pain [146]. Although the exact mechanism of action is not yet fully understood, evidence suggests that continuous use of capsaicin (4–6 times daily for 4–8 weeks) acts as an agonist of the TRPV1 receptor on sensory nerve fibers and prevents the depletion of neuropeptides, thus preventing the transmission of pain and itching, desensitizing the area [147]. Due to its pharmacological action, capsaicin is used to treat various painful conditions and disorders, including chronic rheumatic pain, postherpetic neuralgia, painful diabetic neuropathy, and osteoarthritis [148]. Capsaicin can also be used in patients with bladder hyperactivity to improve bladder capacity and reduce incontinence. Capsaicin has several benefits: it can protect the stomach from gastritis triggered by nonsteroidal anti-inflammatory drugs, relieves postoperative nausea, vomiting and sore throat, and provides relief for patients suffering from pruritus associated with renal failure or cardiac ischemia [124].

## 6 Pharmacological benefits

### 6.1 Analgesic effect

Capsaicin helps relieve pain and discomfort in the body from neuralgia, rheumatoid arthritis, and diabetic neuropathy [149]. People are sensitive to capsaicin, which causes burning, stinging, or itching sensations [150]. However, capsaicin ultimately eliminates pain by raising intracellular Ca^2+^ levels, desensitizing nociceptor fibers, and weakening the pain signaling pathway [151,152]. Although humans are mildly sensitive to capsaicin, which initially causes burning, stinging, or itching sensations, capsaicin ultimately relieves pain by increasing intracellular Ca^2+^ levels, resulting in desensitization of nociceptor fibers and weakening of the pain signaling pathway [[150], [151], [152]]. Subcutaneously injected capsaicin may have analgesic effects in many types of pain by first effectively activating the transient receptor potential vanilloid 1 (TRPV1) receptor [153]. Remarkably, 1 % capsaicin pretreatment inhibited the expression of pain after a plantar incision, suggesting that capsaicin has a preventive analgesic effect [154].

### 6.2 Antioxidant activity

Chili is known to contain a considerable amount of antioxidants due to its pungency [155]. Several *in-vitro* models, including rat liver mitochondria, soybean phosphatidylcholine liposomal membranes, and human erythrocytes, have been used to investigate the inhibitory effect of capsaicin on lipid peroxidation [27,156]. This led to the discovery that capsaicin can reduce ferrous-induced lipid peroxidation by binding to ferrous and ferric ions and preventing the redox cycling of iron in rat brains [157]. The antioxidant characteristic of capsaicin is attributed to its phenolic component, although mechanistic studies contend that C7-benzyl carbon exhibits antioxidant and free radical-suppressing properties due to the formation of active oxygen species, rather than the phenolic group [7,45,55].

### 6.3 Antimicrobial activity

Capsaicin has been known for centuries for its antibacterial properties [158]. Historical records show that both hot and cold extracts of cayenne pepper have been used to treat various infections and have proven effective against bacteria such as *Streptococcus pyogenes*, *Bacillus cereus*, *Bacillus subtilis*, *Clostridium sporogenes*, and *Clostridium tetani* [[55], [159]]. The alcohol in the capsicum fruit has strong properties against both gram-positive and gram-negative bacteria, as well as fungi [[160], [161], [162]]. Capsaicin is responsible for the destruction of the microbial membrane, which leads to the death of the microbes [163,164].

### 6.4 Anti-inflammatory activity

Capsaicin, a capsaicinoid that relieves pain, has attracted the most research interest [165]. Although capsaicin causes inflammation due to nerve stimulation, it is often used in topical gels and patches for pain relief due to its anti-inflammatory properties [17,166,167]. The production of pro-inflammatory mediators and subsequent activation of the TRPV1 channel are known to be associated with the anti-inflammatory effects of capsaicin [168]. TRPV1, a non-selective cation channel, is triggered by a variety of stimuli, including chemical compounds and elements such as unpleasant heat, proton, and vanilloids [[169], [170], [171]]. Studies either deleting the TRPV1 gene or "knocking down" TRPV1 using RNA interference have shown that TRPV1 plays a significant role in pain perception [172,173].

Since TRPV1 antagonists provide a new paradigm in pain management since it is anticipated that they would reduce pain perception by inhibiting undesirable endogenous substances that activate TRPV1 [174,175]. Unlike other natural irritants, capsaicinoids, such as capsaicin or dihydrocapsaicin, cause a prolonged refractory condition known as desensitization after first activating sensory neurons [176,177]. Recent research on anti-inflammatory drugs has also revealed that capsaicin releases endogenous somatostatin, which protects the retina from damage induced by ischemia and reperfusion [178].

### 6.5 Anti-cancer properties

Capsaicin exhibits diverse pharmacological properties, including antigenotoxic, antimutagenic, and anticarcinogenic effects. Nevertheless, conflicting studies suggest its potential role as a tumor promoter, mutagen, and carcinogen, calling capsaicin a “double-edged sword.” Some potential studies on the carcinogenic effect of capsaicin *in-vivo* have been reported. For example, Chanda et al. (2007) evaluated the dermal carcinogenic potential of capsaicin in Tg.AC transgenic mouse model where the analysis revealed that mice treated along with tetradecanoylphorbol-13 acetate (TPA) developed some dermal mass growth [179]. Furthermore, Lui et at., (2012) also found that low concentrations (between 0.1 and 10 μM) of capsaicin can persuade tumor cell growth and migration in HCT116 cells by upregulating the expression of tumor-associated NADH oxidase (tNOX) [180]. Moreover, *in-vivo* research conducted on mice demonstrated that capsaicin stimulates the growth of gastrointestinal tumors in mice [181]. Comprehensive case-control research revealed that capsaicin can promote the growth of cancer cells in those who like spicy food [182] such as colorectal carcinoma [180], and hepatocellular carcinoma [183].

Although prooncogenic impacts are being mentioned in many literatures, these studies' applicability is debatable, and more investigation is required to validate the findings. Meanwhile, in a 2020 research, capsaicin was shown to be safe to eat in large dosages since it did not affect the carcinogenesis development of a rat model of preneoplastic colon cancer [184]. The synergistic anticancer effects of capsaicin alongside other drugs are well-documented. By increasing nitric oxide (NO) in a p53-dependent manner, capsaicin together with resveratrol induced apoptosis. Combining dietary phytoestrogen genistein with capsaicin demonstrated synergistic anticancer behavior by modifying AMP-activated protein kinase (AMPK) and cyclo-oxygenase 2 in breast cancer cells [185]. Clark et al. (2015) reported that capsaicin and 3,3′-diindolylmethane, a significant *in-vivo* metabolite of indole-3 carbinol found in tons in cruciferous vegetables, contribute to stimulate apoptosis in colorectal cancer by modifying the transcriptional activity of nuclear factor kappa B, *p53*, and target genes that regulate apoptosis [186].

Because capsaicin inhibits the Notch signaling pathway in breast cancer stem cells, it affects the viability of cancer stem cells [187]. Cellular proliferation is a crucial marker for cancer prevention and is well acknowledged to have a significant part in multistage carcinogenesis. Dihydrocapsaicin and capsaicin have been shown to reduce cellular metabolic activation, induce cycle arrest, and/or induce apoptosis to impede the proliferation of a variety of immortalized or malignant cell lines [[188], [189], [190], [191], [192]]. The findings suggested that the both receptor-free direct pathway and the receptor-dependent indirect pathway are both implicated in the activation of cellular death by capsaicin or dihydrocapsaicin [193]. In the direct mechanism, capsaicin causes apoptosis by interacting with caspases, especially caspases 1 and 3. Contrarily, the indirect pathway requires capsaicin to interact with TRPV-1, which causes a rise in intracellular calcium and, as a result, the appearance of early and late signs of apoptosis [193]. In conclusion, capsaicin could alter the level of expression of genes and enzymes responsible for cancer cell proliferation, cell cycle arrest, signal transduction, apoptosis, and metastasis, hence exhibiting considerable anticancer activity [96].

### 6.6 Anti-obesity effect

Given the significant increase in obesity over the last decade, tools and techniques for weight loss and maintenance are attracting significant attention worldwide due to the significant threat to public health. Obesity often arises from an imbalance between excessive energy intake and insufficient energy expenditure, which leads to various metabolic problems such as diabetes, insulin resistance, fatty liver disease, and cardiovascular disease, among others [20]. There is a wealth of evidence to support the notion that capsaicin does indeed possess anti-obesity properties [[194], [195], [196]]. Capsaicin stands out as a predominant strategy, demonstrating its ability to inhibit adipogenesis and increase lipid oxidation in adipocytes. It regulates hypothalamic satiety, suppresses appetite by affecting ghrelin, prevents weight gain by upregulating uncoupling protein 2 (UCP2) and uncoupling protein 3 (UCP3), promotes thermogenesis, and maintains metabolic balance by influencing the gut microbiota [[194], [195], [196]]. Capsaicin decreased the expression of CCAAT/enhancer binding protein (C/EBPα), peroxisome proliferator-activated receptor γ (PPARγ), and leptin and increased the expression of PPARα, UCP2, and adiponectin in 3T3-L1 adipocytes, thereby inhibiting the differentiation, proliferation and lipogenesis of preadipocytes [197]. Capsaicin fosters the browning of adipose tissue and prevents high-fat diet-induced obesity in mice by enhancing sirtuin-1 expression, elevating intracellular Ca^2+^ levels, and activating TRPV1 channels [198]. In another animal study involving obese diabetic mice, capsaicin altered the composition of the gut microbiota, raised plasma and ileum levels of glucagon-like peptide-1 (GLP-1), and stopped the increase in blood glucose and insulin levels, ultimately improving glucose homeostasis [199,200]. To explain how capsaicin combats obesity at a molecular level, Joo et al. (2010) utilized a specialized proteomic method to study the protein modifications triggered by capsaicin treatment in the white adipose tissue (WAT) of rats demonstrated that protein related thermogenesis and lipid metabolism were altered upon capsaicin (10 mg/kg) treatment in WAT decreased 8 % body fat. Moreover, levels of vimentin, peroxiredoxin, and NAD(P) H:quinone oxidoreductase 1 (NQO1) were observed considerably lower (>2-fold), despite flavoprotein and aldo-keto reductase increased upon capsaicin treatment [195]. Given the remarkable changes in proteins related to thermogenesis and lipid metabolism after capsaicin treatment, it is clear that capsaicin plays a central role in the control of energy metabolism [201].

### 6.7 Cardiovascular benefits

Researchers found that capsaicin had beneficial effects on the cardiovascular system [201], including improving myocardial I/R injury in animal models [202], protecting rats from doxorubicin-induced cardiotoxicity [203], lowering the prevalence of cardiovascular diseases by inhibiting platelet aggregation and the activity of clotting factors [204], and shielding cardiometabolic organs from dysfunction [70]. Due to the synergistic effects of capsaicin and dihydrocapsaicin in inhibiting *in-vitro* platelet aggregation and thromboxane formation, capsaicin aids in reducing the risk of heart disease [96,205]. Although the mechanism responsible for capsaicin's effect on platelet aggregation is not clear, reports have shown that the anti-hemostatic property of capsaicin is TRPV1-independent. In the same study, it was hypothesized that capsaicin could penetrate the plasma membrane of platelets and alter the fluidity and/or ionic permeability of the membrane [206]. Nevertheless, the presence of TRPV1 in human platelets suggests that capsaicin triggers calcium release from intracellular platelet stores and subsequently contributes to ADP- and thrombin-induced platelet activation [207]. Therefore, further studies are required to demonstrate the anti-hemostatic property of capsaicin and the proposed mechanism.

### 6.8 Gastro-protective effect

Capsaicin-sensitive sensory nerves are widely distributed throughout the gastrointestinal tract and are believed to be essential for protecting the mucosa of the gastrointestinal tract from damaging stimuli and maintaining its integrity [208]. Furthermore, capsaicinoids have demonstrated gastroprotective properties in numerous animal models of gastrointestinal mucosal damage caused by substances such as hydrochloric acid, ammonia, ethanol, aspirin, or indomethacin [209,210]. The effect of capsaicinoids on the gastrointestinal mucosa can vary significantly, with both positive and negative consequences depending on the dosage and duration of drug intake. High doses of capsaicinoids tend to cause neurotransmitter depletion and damage to capsaicin-sensitive sensory nerves, potentially leading to negative effects on the digestive system [211]. However, lower doses may improve basal blood flow to the gastric mucosa, increase gastric mucus secretion, and accelerate healing of gastric epithelial tissue, all of which contribute to the protection and defense of the gastrointestinal system [212]. Capsaicin is widely employed as a research tool in gastrointestinal physiology, pathology, and pharmacology, owing to its dual effect on sensory neurons, which can be either sensitized or desensitized, although its clinical applications are limited [209].

## 7 Future perspective and conclusion

This review highlights the growing interest in natural compounds, particularly those from the pepper plant, as alternatives to synthetic ingredients in the food, pharmaceutical and cosmetic industries. The key findings suggest that capsaicinoid and carotenoids from peppers not only enhance the sensory properties of products but also offer significant health benefits, including antioxidant, anti-inflammatory, and antimicrobial effects. However, the full potential of these compounds is yet to be realized due to challenges in standardization, stability, and regulatory approval.

The future of pepper-derived products relies heavily on addressing these challenges. Standardizing the bioactive compounds for consistent color, flavor, and biological activity is essential for unlocking their full market potential. Moreover, the development of "green" extraction methodologies that minimize environmental impact and optimize yield is crucial for sustainable production.

As consumer preference shifts towards natural ingredients, an industry centered on functional compounds and natural therapeutics is expanding. Integrating these natural substances into daily diets can enhance the organoleptic properties of food, extend product shelf life, and bolster human health. The drive to standardize these extracts for use in dermatological and cosmetic applications highlights the urgency for clear regulatory frameworks that guarantee safety and effectiveness before market release.

Future research should prioritize elucidating the mechanisms by which Capsicum components exert their benefits at the industry and consumer levels. Such studies will shed light on the potential of pepper byproducts as sources of valuable bioactive compounds, thereby ensuring consumer safety and technological advancement. Additionally, thorough epidemiological and clinical research is needed to support health claims, which will facilitate the integration of these compounds into health and wellness products worldwide. Establishing clear regulatory guidelines for the safety and effectiveness of these bioactive ingredients is essential to promote industry innovation and protect consumer interests. Addressing these critical challenges will optimize the application of pepper-derived compounds, leading to the development of natural, effective, and sustainable products across various sectors, thereby advancing both industry and public health outcomes.

## CRediT authorship contribution statement

**Anoth Maharjan:** Writing – original draft. **Bala Murali Krishna Vasamsetti:** Conceptualization, Writing – original draft. **Jung-Ho Park:** Writing – review & editing.

## Consent for publication

All authors agreed to submit this manuscript.

## Data availability statement

All the data are included within the article.

## Declaration of competing interest

The authors declare that they have no known competing financial interests or personal relationships that could have appeared to influence the work reported in this article.
